# Usability of the GAIMplank Video Game Controller for People With Mobility Impairments: Observational Study

**DOI:** 10.2196/38484

**Published:** 2023-01-10

**Authors:** Laurie A Malone, Christen J Mendonca, Sangeetha Mohanraj, Samuel R Misko, Joseph Moore, James Michael Brascome, Mohanraj Thirumalai

**Affiliations:** 1 School of Health Professions The University of Alabama at Birmingham Birmingham, AL United States; 2 Engineering and Innovative Technology Development The University of Alabama at Birmingham Birmingham, AL United States

**Keywords:** exergaming, physical disability, equipment design, video gaming, physical activity, exercise, wheelchair

## Abstract

**Background:**

Replacing sedentary behaviors during leisure time with active video gaming has been shown to be an enjoyable option for increasing physical activity. However, most off-the-shelf active video gaming controllers are not accessible or usable for individuals with mobility impairments. To address this requirement, a universal video game controller (called the GAIMplank) was designed and developed.

**Objective:**

This study aimed to assess the usability of the GAIMplank video game controller for playing PC video games among individuals with mobility impairments. Measures of enjoyment, perceived exertion, and qualitative data on the user experience were also examined.

**Methods:**

Adults (aged 18-75 years) with a mobility impairment were recruited to participate in a single testing session in the laboratory. Before testing began, basic demographic information, along with minutes of weekday and weekend physical activity, minutes of weekday and weekend video game play, and video game play experience were collected. The GAIMplank was mapped to operate as a typical joystick controller. Depending on their comfort and functional ability, participants chose to play seated in a chair, standing, or in their own manual wheelchair. Leaning movements of the trunk created corresponding action in the game (ie, lean right to move right). The participants played a total of 5 preselected video games for approximately 5 minutes each. Data were collected to assess the usability of the GAIMplank, along with self-efficacy regarding execution of game play actions, rating of perceived exertion and enjoyment for each game, and overall qualitative feedback.

**Results:**

A total of 21 adults (n=15, 71% men; n=6, 29% women) completed the usability testing, with a mean age of 48.8 (SD 13.8; range 21-73) years. Overall, 38% (8/21) of adults played while standing, 33% (7/21) of adults played while seated in a chair, and 29% (6/21) played in their own manual wheelchair. Scores from the System Usability Scale indicated above average (74.8, SD 14.5) usability, with scores best for those who played seated in a chair, followed by those standing, and then individuals who played seated in their own wheelchairs. Inconsistencies in the responsiveness of the controller and general feedback for minor improvements were documented. Rating of perceived exertion scores ranged from light to moderate intensity, with the highest scores for those who played seated in a chair. Participants rated their experience with playing each game from above average to very enjoyable.

**Conclusions:**

The GAIMplank video game controller was found to be usable and accessible, providing an enjoyable option for light-to-moderate intensity exercise among adults with mobility impairments. Minor issues with inconsistencies in controller responsiveness were also recorded. Following further development and refinement, the next phase will include a pilot exercise intervention using the GAIMplank system.

## Introduction

### Background

Physical activity is an important component of a healthy lifestyle. A myriad of health benefits, both physical and psychological, can be achieved with daily physical activity [1]. Physical activity options, however, are limited for people with mobility impairments. Issues with accessibility and transportation are experienced by many individuals wanting to fulfill their daily physical activity needs. Fitness center parking, inaccessible entrances, limited staff knowledge on inclusive programming, and cost of membership and transportation are some of the limiting factors [2]. Equipment such as a stationary arm ergometer can provide a means to exercise at home but may be seen as boring and difficult to adhere to by many [3]. In an effort to combat the tedium of routine exercise, active video gaming (AVG) has become popular.

Replacing sedentary behaviors during leisure time with AVG has been shown to be an enjoyable option for increasing the amount of physical activity acquired each week [4-12]. The promise of AVGs is particularly appealing for people with disabilities, given the high rate of inaccessible features in the built environment and the difficulty in finding fun and engaging activities [5-8]. Because AVG systems are relatively affordable, they also hold promise as a scalable product for promoting improved health outcomes and higher levels of physical activity and fitness among people with disabilities [13]. Several AVG systems have been in the market for the last 2 decades; however, most are not adapted to suit the needs of individuals with mobility impairments. Using a mixed methods study, the usability of a somatosensory square dance system for older adults was examined [14]. The system was developed to support a popular fitness activity and to eliminate some of the common barriers (risk of injury, noise, and space) experienced by older adults. The system was well received by the participants, providing them with a fun fitness activity that could be safely performed indoors in a private space. The participants included a group of older adults (aged 55-68 years); however, specific adaptation for individuals with mobility impairments was not the focus and therefore was not considered. Another study examined the usability of an AVG platform for older adults with various physical and sensory impairments residing in long-term care homes, with 85% having a mobility impairment [15]. After several rounds of usability testing with residents, staff, and family, the feedback indicated that the system was an enjoyable way to engage in physical activity. Although efforts are being made, there continues to be a pressing need to make AVG controllers accessible to people who are unable to stand for long periods, cannot stand on a small platform because of poor balance or extreme obesity, or those who use a wheelchair for all daily activities.

Our engineering design and development team, with the Rehabilitation Engineering Research Center on Interactive Exercise Technologies and Exercise Physiology for People with Disabilities, previously built a proof-of-concept (PoC) wheelchair-accessible adapted gaming controller for the Wii system [16] and our research team tested its usability rating compared with the commercial off-the-shelf (OTS) balance board controller provided with the product [17]. Participants with mobility impairments found the wheelchair-accessible version to be more usable compared with the Wii OTS board (*P*<.01). The mean usability scores were 71.7 (SD 18.03) for the adapted controller and 32.1 (SD 36.72) for the Wii OTS board (higher scores reflect greater usability). In addition, a significant negative correlation (*r*=0.692; *P*<.001) was found between lower extremity function and system usability scores, indicating that for participants with poorer lower extremity function, the adapted board was perceived as more usable. Furthermore, participants reported activity using the adapted board to be enjoyable, achieving light-to-moderate intensity exercise [18].

As a next step, the aim was to transition from a PoC gaming controller to the performance of highly targeted proof-of-product (PoP) activities for the design of a new wheelchair-accessible AVG controller using the expertise and input of engineers, product designers, potential users, and other stakeholders from the community. The goal was to convert from a Wii-only interface to a universal controller for use with all games available to play on a PC.

### Study Objectives

Following the design and development process, this study aimed to assess the usability of a wheelchair-accessible GAIMplank video game controller for individuals with mobility impairments. Measures of enjoyment, perceived exertion, and qualitative data on user experience were also collected.

## Methods

### PoP Development Process

To begin our PoP development effort, approximately 30 semistructured interviews were conducted with end users in various market segments to discover use cases that would be used to drive design features, functions, and capabilities. The physical therapy clinic market segment was found to be lacking in financial resources for investment into technology for nonreimbursable activities such as AVG. The market segment for inpatient rehabilitation and retirement facilities and communities was found to have the financial resources for recreational activities but lacked robust end user interest. The universal video game accessory market was found to be much more robust in terms of interest for nontraditional game controllers to facilitate intuitive player movement in virtual reality environments as well as to encourage AVG. Effective entry into this market requires the game controller to (1) be easily transportable and stowable, (2) have a purchase price comparable with other high-end game controllers (<US $300), and (3) be universally compatible with all major gaming platforms.

**Figure 1:**
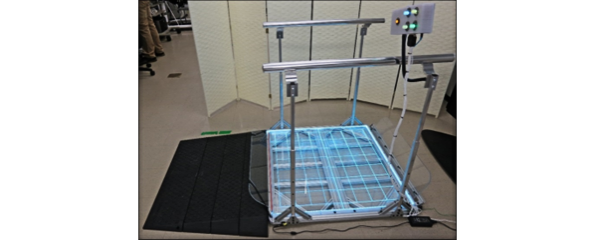
Proof-of-concept balance board prototype.

The design process began based on the PoC prototype developed previously (Figure 1), which used a custom-built aluminum frame, user interface, and load cell sensors combined with repurposed internal electronics from a deconstructed Wii Fit Board to allow for wireless connection to Wii game consoles. On the basis of the market study results, the preliminary PoP design effort targeted 3 areas for improvement, including reducing the weight of the balance board to <6.8 kg, integrating custom electronics for universal compatibility with all major gaming platforms, and optimizing design for low-cost manufacturing.

**Figure 2:**
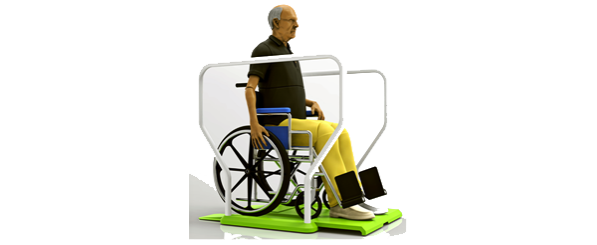
Early proof-of-product conceptual design for inpatient rehabilitation market.

A redesign of the PoC balance board platform began with the determination of the critical dimensions for use by standing and seated users while also minimizing the footprint and structural support of the weightbearing structure. Early conceptual designs featured novel solutions to these design challenges through the integration of handrails, footrests, and ramps into the platform design (Figure 2). After evaluating a series of finite element models and simplified prototypes, a resin-infused sandwich composite was the material selected for use to reduce the weight of the balance board while maintaining the stiffness necessary to redistribute the user’s weight to a minimum number of load cells.

The redesign of the PoC balance board electronics began with the selection of the embedded processor, load cell measurement integrated circuits, and wired and wireless communication modalities. The user interface components such as the sensitivity adjustments (overall, front and back, right and left) were upgraded from the PoC’s buttons and knobs to an Android app that can easily provide access to any number of different controller settings and functions to optimize the user’s game play experience. A Raspberry Pi Zero was implemented as the primary processor facilitating Bluetooth connection to an Android tablet as well as allowing remote access to data and programming via Wi-Fi. The USB Human Interface Device periphery connection to PCs and gaming console adapters was facilitated using an ARM Cortex-M4 microcontroller configured as a generic joystick device. Load cell measurements were performed using dedicated low-noise 24-bit analog-to-digital microchips.

**Figure 3:**
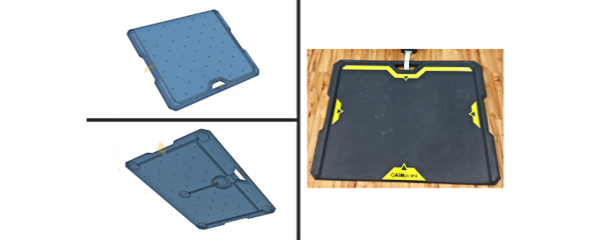
Final proof-of-product design and prototype for the GAIMplank video game controller. Photos on the left show the top view (upper left) and bottom view (lower left) of the board before the final coating and graphics were applied (right).

The final design of the GAIMplank balance board platform (Figure 3) features rims on 3 sides of the board that serve as a physical barrier at the edge of the board. A downward tapered edge along the back of the board, and a slimline ramp, allowed for easy roll-on access (eg, if the user is in a wheelchair). The GAIMplank platform also features yellow high-visibility graphics that provide the user with a sense of their alignment on top of the board. The weight capacity of the GAIMplank platform is ≤270 kg, which is sufficient to support a wide range of users.

**Figure 4:**
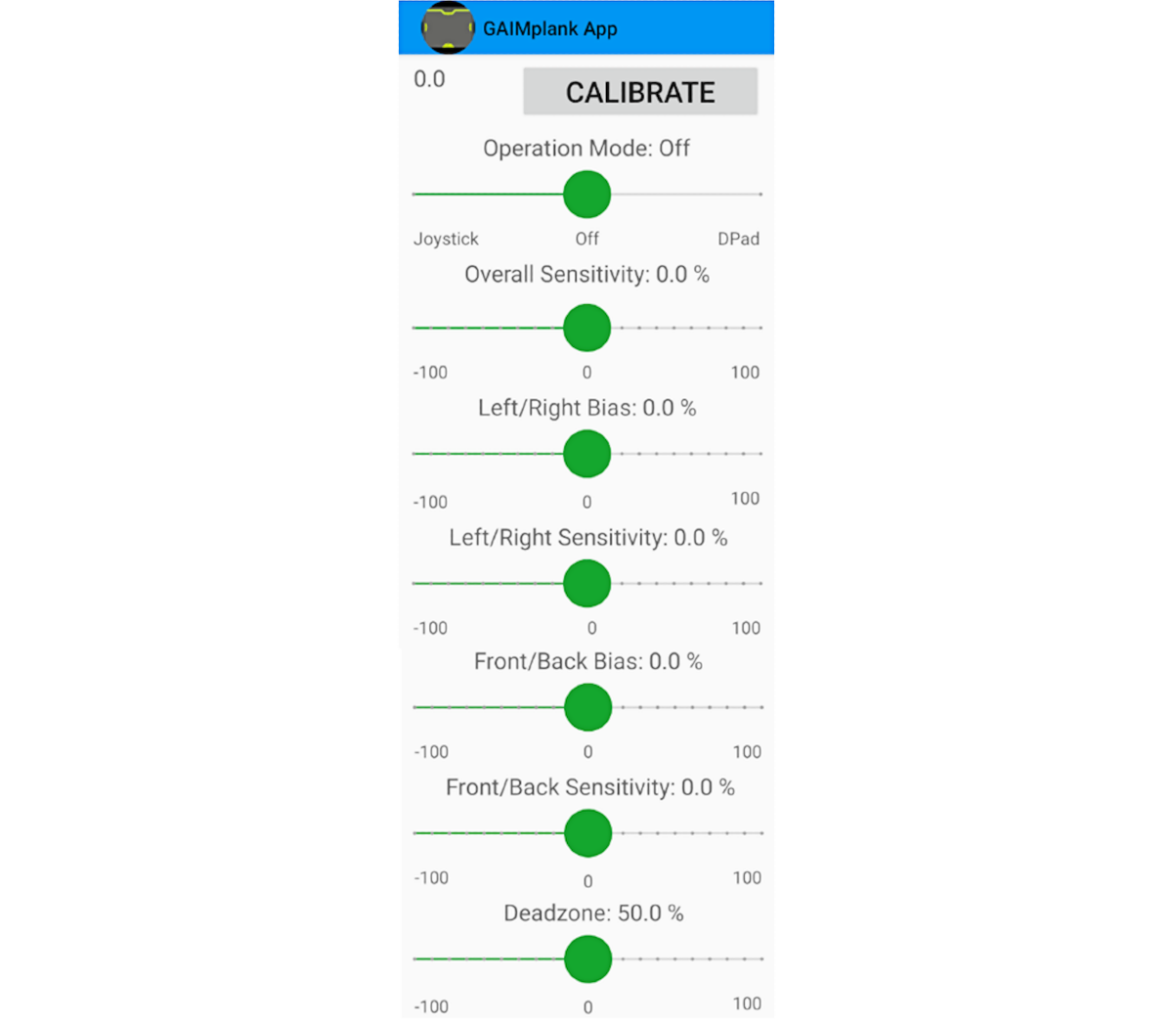
Picture of the GAIMplank app with slider bars used to calibrate the device; select mode of play; and adjust sensitivity, bias, and dead zone area.

The final design of the GAIMplank Android App features real-time bidirectional information flow and access to a large range of customizable settings for real-time adjustments (Figure 4) to the measured load cells’ bias, dead zones, and sensitivity, as well as to allow for on-demand calibration of the GAIMplank’s output based on a player’s measured weight and range of movement and game play mode (joystick vs directional pad).

**Figure 5:**
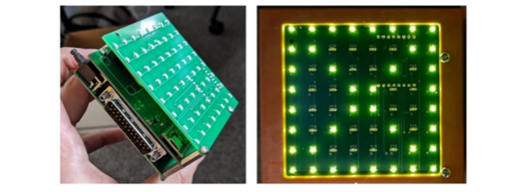
Proof-of-product design of the GAIMplank electronics stack.

The final design of GAIMplank electronics includes a custom printed circuit board (PCB) stack and an LED matrix display (Figure 5). The need for this display was discovered during the early developmental testing of prototypes with 3 volunteers recruited from local video gaming clubs, whereby they required instant feedback to build confidence that the board was indeed responding accurately and quickly to their movements during game play. This display is also used to guide the user through the required movements during calibration (step off, step on, move forward and backward, and move left and right). The PCB stack includes a Raspberry Pi as the central processor for the device, controlling wireless and wired communication, the LED matrix, and the measurement of the position of the user’s center of balance at a rate of approximately 50 Hz. The stack also provides robust fixturing for the 22-pin D-Sub connector, which is used to connect to the load cells embedded in the platform via a flat flexible cable, as well as for the mini USB port for a wired connection to the gaming PC for the game console adapter.

To ensure safety during game play, height-adjustable handrails were located on 3 sides of the GAIMplank. In addition, a Microsoft adaptive controller was integrated into the system to facilitate the use of external buttons and triggers used during game play.

### Usability Testing

#### Design and Setting

Data collection for usability testing was performed at the RERC RecTech Exercise Science and Technology Laboratory (University of Alabama at Birmingham). For the purposes of this study, participants came to the laboratory for a single visit, which lasted for approximately 60-90 minutes.

#### Ethics Approval

All study procedures were approved by the institutional review board at the University of Alabama at Birmingham (IRB-300003265).

#### Participant Recruitment

Participants were recruited via flyers and word of mouth at a local community health and fitness center for individuals with physical disabilities and chronic health conditions. To assess the usability of the GAIMplank for different play styles (sitting in a chair, personal wheelchair, and standing), recruitment was stratified. Sample size estimates were based on identifying common usability barriers and issues specific to the 3 game play styles. According to Cazañas et al [19], a sample of 17 individuals would reasonably identify 80% of common problems in the system, with groups of 4 to 9 sufficient to identify problems specific to the mode of play. Additional participants were recruited to account for modest attrition.

Inclusion criteria for potential participants were (1) age 18-75 years; (2) a self-reported lower extremity mobility disability (eg, spina bifida, cerebral palsy, muscular dystrophy, >1 year after spinal cord injury, multiple sclerosis, stroke, or limb loss) with partial or full use of the upper extremities; (3) mobility impairment (ie, gait deviation and balance issue) or use of an assistive device (manual wheelchair, walker, crutches, and canes) for balance or mobility purposes; (4) ability to use arms for exercise; and (5) body weight <180 kg. Exclusion criteria included (1) significant impairment in visual acuity that prevents seeing a 52” television screen to follow exercise, (2) cardiovascular disease event within the past 6 months, (3) severe pulmonary disease or renal failure, (4) current pregnancy, (5) ongoing exacerbation of a health condition, and (6) other conditions that would interfere with intervention or testing procedures. Following the distribution of a flyer, the project recruitment coordinator answered calls from or met interested individuals. If contacted, the recruitment coordinator reviewed the inclusion and exclusion criteria using a screening form to determine whether they were eligible to participate.

### Testing Session

Upon arrival at the laboratory, all study procedures were reviewed with the participant. After being given an opportunity to ask questions, the participants provided informed consent. Before the testing began, basic demographic information, along with information regarding minutes of weekday and weekend physical activity, minutes of weekday and weekend video game play, and video game play experience were collected. All procedures for data collection and video game operation were reviewed with the participants before game play. The participants were then settled at the GAIMplank station. Before playing the first game, a calibration procedure was conducted by following a pattern of movements as indicated on the LED matrix.

**Figure 6:**
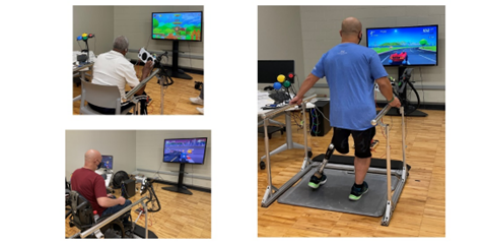
Participants standing (right), seated in a chair (upper left), or wheelchair (bottom left) engaging in active video gaming using the GAIMplank controller. Game play movement was controlled by shifting the body weight or leaning forward, back, right, left as needed. External buttons to activate actions such as jump or shoot could be configured in various formations on a flexible arm within the player’s reach. A trigger button used for acceleration or shooting could be held in the hand or placed on the external button pad.

The GAIMplank was mapped to operate as a typical joystick controller. Leaning movements of the trunk created corresponding actions in the game (ie, lean forward to move character forward and up, lean right to move character right; Figure 6). The participants played a total of 5 preselected video games. The game Feather, a slow-paced flying game, was played first by all participants to introduce them to the movements required for operating the GAIMplank. The order in which the remaining 4 games were played was randomized. To minimize offensive content, the games had an Entertainment Software Rating Board score of E (Everyone), E+10, or Teen. The description of each game played and movements required is provided in Table 1. Each game was played for approximately 5 minutes.

**Table 1:** Description of games played and movements required.

Game	Genre	Game description	Trunk leaning movements required	External buttons used
*Feather*	Flight; exploration	Relaxing flight to explore a scenic nature landscape	Left, right, forward (down), backward (up), diagonal	None
*Let’s Go Nuts*	2D platformer; action	Jump, avoid danger, and collect nuts as you progress through the levels	Left, right	1 large button for jump
*Horizon Chase Turbo*	Racing; sports; arcade	Arcade racing games with various cars and tracks to select from	Left, right	Variable trigger for car acceleration
*PAC-MAN* *Championship Edition DX+*	Arcade; action	Move through the maze in this classic ghost chomping arcade game	Left, right, forward (up), backward (down)	1 large button to scare away the ghosts
*Stardust Galaxy* *Warriors*	Shooter; action	Soaring through space using various weapons to combat asteroids and enemy ships	Left, right, forward (up), backward (down), diagonal	Up to 4 buttons for weapon use

### Measures

#### Quantitative Measures

The participants’ responses to a series of survey measures were collected during the session. Descriptive information was collected regarding enjoyment and current level of physical activity and video gaming. Before beginning game play, participants were shown a short video demonstrating a player using the system under 3 different conditions: sitting in a 4-legged chair, sitting in a wheelchair, and standing.

Given that higher self-efficacy is correlated with higher exercise participation [20], positive physical activity behaviors [21], AVG enjoyment [22], exercise adherence [23], exercise duration [24], and AVG approval [25], participants were asked to rate their level of task self-efficacy after watching the video. This scale (Multimedia Appendix 1) was developed based on the recommendations of Bandura et al [26] and consisted of 6 items that best represent the dimensions of the task. These dimensions equally represent the steps to the Videogame Interaction Model proposed by Yuan et al [27], which include receiving stimuli, determining response, and providing input. Each item on the scale was scored on an 11-point Likert scale from no certainty (0) to absolute certainty (10) in performing the 6 dimensions related to game play (maintaining focus, seeing and hearing game information, reacting fast, determining movement strategies, coordinating movements, and moving well). The gradations of scoring for the scale were consistent with expert recommendations for measuring self-efficacy [26]. Participants rated their self-efficacy again at the end after playing all 5 games. An average score for the 6 items was calculated, with a higher score indicating greater self-efficacy in their ability to execute game play actions.

After playing each game, participants were asked to rate their level of perceived exertion (rating of perceived exertion [RPE]) and enjoyment. For RPE, the 0 (extremely easy) to 10 (extremely hard) point adult OMNI scale was used [28]. For enjoyment, participants were presented with a visual analog scale, with anchors from “Not at All Enjoyable” on the left to “Extremely Enjoyable” on the right, and were asked to mark a spot on the line to represent their enjoyment.

After finishing all game play, participants were asked to complete 2 usability surveys. The participants’ perceived usability of the adapted video gaming controller was assessed using the System Usability Scale (SUS) and Health-IT Usability Evaluation Scale (HITUES). These 2 scales were selected to obtain general system usability data (SUS) in addition to usability data specific to AVG play using the GAIMplank (HITUES).

For the SUS, respondents answered 10 questions using a 5-point Likert scale ranging from strongly disagree (1) to strongly agree (5). The SUS is widely used as a reliable and valid measure of a system’s usability [29-31]. Reliable results can be obtained with small samples [32], and the tool is applicable to a wide range of systems [29,33]. Using the recommended scoring guidelines, the SUS produces a score ranging from 0 to 100 [31,34]. An average SUS score of 68 corresponds to a percentile ranking of 50%, so a score >68 would be considered above average, and a score <68 is considered below average [30,33]. Adjective ratings, to help with interpretation, have been found to correlate well with the SUS scores [35].

The HITUES consisted of 20 items rated on a 5-point Likert scale ranging from strongly disagree (1) to strongly agree (5). The HITUES has 4 sections with questions to address impact, perceived usefulness, perceived ease of use, and user control. Furthermore, the HITUES allows questions to be tailored to the system under study. For the purposes of this study, each question was phrased to assess the use of the “adapted gaming board” for “active video gaming” (Multimedia Appendix 2). Studies have demonstrated the reliability and validity of the HITUES [36-38]. One recent study suggested a cutoff score of 4.32 to indicate acceptable usability, but further validation studies are required to confirm this [39].

#### Qualitative Measures

Completion of the usability surveys was then followed by collection of feedback data via surveys and semistructured interviews. The questions for both were written with input from members of our engineering team to obtain feedback regarding current usability and to identify issues that needed to be addressed in future iterations and to help in the next phase of the GAIMplank development. Initially, the participants provided written responses to a series of multiple choice, yes or no, and short answer questions to obtain feedback regarding the system (Multimedia Appendix 3). Participants completed these questions on their own. After the first 8 participants, the research staff began conducting semistructured interviews with participants to facilitate more in-depth responses. Using an interview guide (Multimedia Appendix 4), the feedback questions were asked by a member of the research team in a semistructured interview format, with simple prompts (eg, please tell me more) given when appropriate. The interviews were audio recorded and later transcribed.

## Results

### Overview

A total of 21 adults (n=15, 71% men and n=6, 29% women) completed the usability testing. The participant characteristics are provided in Table 2. The mean age of the participants was 48.8 (SD 13.8; range 21-73) years, with 57% (12/21) White, 38% (8/21) Black, and 5% (1/21) Asian. All participants had a mobility impairment resulting from a physical disability, including stroke (9/21, 43%), spinal cord injury (3/21, 14%), amputation (3/21, 14%), cerebral palsy (2/21, 9%), spina bifida (2/21, 9%), or other (2/21, 9%). The primary modes of mobility included walking without an assistive device (7/21, 33%), cane (6/21, 29%), prosthetic leg (1/21, 5%), rollator walker (1/21, 5%), and manual wheelchair (6/21, 29%). The participants played in 1 of the following 3 ways: standing (8/21, 38%), seated in a 4-legged chair (7/21, 33%), or seated in their own manual wheelchair (6/21, 29%).

Participants reported enjoyment of leisure time physical activity, were physically active, and varied in their level of video game play (Table 3). All but 2 participants were aware of AVG. The 2 most preferred devices for playing video games included video game consoles and cell phones.

The usability data for each individual participant are reported in Table 2, with the summary group scores in Table 4. Subscale scores (usability and learning) for the SUS usability tool are also reported. The overall SUS score for all participants was 74.8 (SD 14.5), suggesting above average usability for the GAIMplank. On the basis of the adjective ratings for the SUS scale as provided by Bangor et al [35], the participant scores suggested *good* usability. When broken down by play style group, the average SUS scores indicated that the usability was best for those who played while seated in a chair, followed by those who played standing, and lowest for individuals who played while seated in their own wheelchairs. A similar pattern of scores was seen with the HITUES scale, with an average score of 4.3 (SD 0.3) for all participants. The HITUES scores can range from 1 to 5, with a higher score indicating greater usability.

RPE and enjoyment scores are reported for each game by play style group in Table 5. RPE scores ranged from light to moderate intensity, with the highest scores for those who played while seated in a chair. Participants rated their experience with playing each game from above average to very enjoyable.

The task self-efficacy questions were used to gauge the participants’ self-efficacy in performing certain video game play tasks using the GAIMplank system. Above average scores were reported by all 3 groups for each task (Table 6). It appears that one exposure to video game play with the GAIMplank did not diminish their task self-efficacy, and the participants felt confident in their ability to complete game video play tasks using the GAIMplank system.

**Table 2:** Participant characteristics and usability scores presented by play style grouping (wheelchair, seated, and standing).

ID	Age (years)	Sex	Condition	Primary, secondary assistive device	GAIMplank play style	Enjoy video game play (1-5)	Video game play (minutes per week)	SUS^a^ total score	HITUES^b^ score
4	38	Male	Spinal cord injury	Wheelchair	Wheelchair	5	320	52.5	4.35
12	52	Male	Spinal cord injury	Wheelchair	Wheelchair	4	480	70	4.3
13	52	Male	Double above-knee and double below-elbow amputation	Wheelchair	Wheelchair	4	210	87.5	4.18
14	39	Male	Spina bifida	Wheelchair	Wheelchair	5	120	40	4.15
20	48	Female	Spina bifida	Wheelchair	Wheelchair	3	0	87.5	4.1
21	58	Male	Spinal cord injury	Wheelchair	Wheelchair	5	2520	62.5	3.6
2	65	Male	Stroke	Cane	Seated	1	0	82.5	4.75
3	73	Male	Stroke	Cane	Seated	4	30	90	4.5
6	70	Male	Stroke	Cane, walker, wheelchair	Seated	5	30	87.5	4.6
7	63	Male	Stroke	Cane, wheelchair	Seated	4	0	x	x
8	41	Female	Stroke	Cane, wheelchair	Seated	2	0	67.5	4.3
10	48	Male	Stroke	None, cane	Seated	5	0	90	4.58
15	63	Male	Spinal cord injury	Rollator	Seated	4	70	80	4.58
1	50	Male	Stroke	Cane, wheelchair	Stand	5	60	62.5	4.8
5	42	Male	Single above-knee amputation	Prosthetic leg	Stand	5	1380	72.5	4.15
9	59	Female	Stroke	Walk	Stand	4	900	75	4.05
11	42	Female	Stroke	None, cane	Stand	5	630	97.5	4.45
16	29	Male	Cerebral palsy	None	Stand	4	320	65	4.15
17	32	Female	Single below-knee amputation	Prosthetic leg, wheelchair	Stand	5	1275	77.5	4.6
18	21	Female	Hydrocephalus, balance issues	None	Stand	4	10	85	4.43
19	40	Male	Cerebral palsy	None	Stand	3	0	62.5	4.15

^a^SUS: System Usability Scale.

^b^HITUES: Health-IT Usability Evaluation Scale.

**Table 3:** Participants’ enjoyment of physical activity, weekly minutes of physical activity, enjoyment of playing video games, and weekly minutes of video game play.

	All (n=21), mean (SD)	Wheelchair (n=6), mean (SD)	Seated in chair (n=7), mean (SD)	Standing (n=8), mean (SD)
I enjoy engaging in physical activity during my leisure time (1=strongly disagree, 5=strongly agree)	4.6 (0.6)	4.5 (0.5)	4.7 (0.5)	4.5 (0.8)
Weekday leisure-time physical activity that increases heart rate (minutes)	259 (177)	263 (230)	248 (171)	263 (163)
Weekend leisure-time physical activity that increases heart rate (minutes)	116 (103)	100 (131)	108 (70)	134 (111)
I enjoy playing video games (1=strongly disagree, 5=strongly agree)	4.1 (1.1)	4.3 (0.8)	3.6 (1.5)	4.4 (0.7)
Weekday video gaming (minutes)	264 (462)	462 (756)	14 (27)	359 (389)
Weekend video gaming (minutes)	138 (219)	204 (296)	4 (11)	213 (230)

**Table 4:** Self-reported GAIMplank usability scores.

Game play style	SUS^a^ overall, mean (SD)	SUS: usability subscale, mean (SD)	SUS: learning subscale, mean (SD)	HITUES^b^, mean (SD)
All players	74.8 (14.5)	75.2 (15.5)	73.1 (24.8)	4.3 (0.3)
Wheelchair (n=6)	66.7 (19.0)	66.7 (19.2)	66.7 (30.3)	4.1 (0.3)
Seated in chair (n=7)	82.9 (8.6)	85.4 (10.8)	72.9 (16.6)	4.6 (0.1)
Standing (n=8)	74.7 (12.1)	73.8 (12.3)	78.1 (27.3)	4.3 (0.3)

^a^SUS: System Usability Scale.

^b^HITUES: Health-IT Usability Evaluation Scale.

**Table 5:** Rating of perceived exertion and enjoyment rating for each game by player group.

Game			Rating of perceived exertion (0-10), mean (SD)		Enjoyment (0-100), mean (SD)
**All players, across games**			4.2 (1.7)		72.3 (16.5)
	Wheelchair	4.5 (1.1)		68.9 (12.4)	
	Seated in chair	5.1 (2.2)		79.5 (21.1)	
	Standing	3.4 (1.5)		69.4 (15.7)	
**Feather**			3.4 (1.9)		62.7 (32.7)
	Wheelchair	3.7 (1.4)		68.5 (23.9)	
	Seated in chair	4.0 (2.2)		69.0 (38.9)	
	Standing	2.6 (1.9)		52.9 (34.3)	
**Let’s Go Nuts**			3.6 (2.0)		76.3 (17.8)
	Wheelchair	4.0 (1.1)		67.3 (15.8)	
	Seated in chair	4.3 (2.9)		78.2 (17.7)	
	Standing	2.7 (1.5)		82.3 (18.9)	
**Horizon Chase Turbo**			4.0 (2.0)		70.1 (28.5)
	Wheelchair	4.0 (1.5)		65.8 (18.0)	
	Seated in chair	5.0 (2.2)		71.3 (45.0)	
	Standing	3.0 (1.9)		72.3 (18.0)	
**PAC-MAN**			4.9 (2.2)		66.3 (24.6)
	Wheelchair	5.3 (2.3)		68.8 (18.2)	
	Seated in chair	5.5 (2.7)		66.7 (36.8)	
	Standing	4.1 (1.8)		64.1 (20.6)	
**Stardust galaxy warriors**			5.2 (1.9)		80.9 (15.2)
	Wheelchair	5.5 (1.4)		74.0 (4.2)	
	Seated in chair	6.0 (2.5)		91.7 (11.7)	
	Standing	4.3 (1.4)		77.9 (19.0)	

**Table 6:** Self-reported task self-efficacy before and after the game play session.

Task self-efficacy question	All (n=21), mean (SD)			Wheelchair (n=6), mean (SD)			Seated in chair (n=7), mean (SD)			Standing (n=8), mean (SD)	
	Before	After	Before		After	Before		After	Before		After
Maintaining focus throughout a 15-minute session	8.8 (1.7)	9.6 (0.9)	9.5 (1.2)		10.0 (0.0)	7.1 (1.9)		8.7 (1.2)	9.8 (0.7)		10.0 (0.0)
Seeing and hearing all the game information	9.4 (1.3)	9.6 (0.9)	9.5 (1.2)		9.7 (0.8)	9.0 (1.9)		9.3 (0.8)	9.8 (0.7)		9.6 (1.1)
Reacting fast enough to choose a next action	7.0 (2.0)	7.7 (1.8)	6.5 (2.3)		7.3 (1.4)	6.1 (1.6)		6.7 (1.9)	8.1 (1.6)		8.6 (1.6)
Determining strategies to move during play	7.0 (2.6)	7.7 (1.8)	5.8 (3.3)		7.0 (1.7)	6.0 (2.2)		6.7 (1.9)	8.6 (1.7)		8.9 (1.4)
Coordinating body movements to carry out a strategy	6.9 (2.5)	7.7 (1.8)	6.2 (3.5)		7.7 (1.0)	6.0 (1.7)		7.7 (1.2)	8.1 (1.8)		7.8 (2.7)
Moving well enough to maintain successful play	7.0 (2.4)	8.1 (2.0)	5.7 (2.4)		7.7 (2.1)	6.6 (2.4)		8.3 (1.2)	8.3 (2.0)		8.3 (2.5)
Overall	7.7 (1.7)	8.4 (1.1)	7.2 (1.8)		8.2 (0.8)	6.9 (1.9)		7.9 (0.8)	8.8 (1.1)		8.9 (1.4)

### Participant Feedback

#### Overview

Feedback data were collected in a written response to multiple choice, yes or no, and open-ended survey questions (6/7, 86% seated and 1/7, 14% standing) or orally via a semistructured interview (5/11, 45% wheelchair; 1/11, 9% seated; and 5/11, 45% standing) conducted by the researchers. Data were missing for 1 survey participant and 2 interview participants.

From the survey feedback, when asked about the ease of mounting and dismounting the gaming board as “relatively simple,” “needs improvement,” or “difficult,” all participants rated both actions as “relatively simple.” When asked whether the gaming board was sturdy, 6 of the 7 participants reported “yes.” All participants indicated that “yes” they were able to determine where best to position themselves on the gaming board and 86% (6/7) reported that “yes” visual cues should be included to locate the central position (on the gaming board). In addition, 86% (6/7) of participants indicated that “yes,” moving their trunk (leaning) provided a responsive input for game control. Additional input functions that the participants would like to see incorporated into the gaming board included visual markers for foot placement, more games, and moving the side handrails closer. When asked to describe their overall experience on the gaming board, comments included “Positive, thank you!” “Very good,” “Something new to keep alert and enjoy,” “Entertaining,” “A lot of fun,” “It was cool to watch my character turn as I leaned left or right,” and “It was great. It was challenging because I am not a gamer, but it was a good challenge.”

Audio recordings of the interviews were transcribed verbatim. The transcripts were then reviewed independently by 2 members of the research team. From the independent reviews, themes were drawn, followed by discussion and review, with a final consensus reached on 5 main themes. These themes included accessibility of the GAIMplank for persons with mobility impairments, usability of the GAIMplank for persons with mobility impairments, overall experience using the GAIMplank, physical activity associated with game play, and suggestions for future iterations.

#### Accessibility of the GAIMplank

This category examined the perceptions of the participants regarding the accessibility of the GAIMplank controller for video game play. Three specific features were discussed, including mounting and dismounting of the GAIMplank, handrails, and accessories. Most participant comments indicated adequate accessibility for video game play. None of the participants reported difficulty in mounting or dismounting the GAIMplank. The handrails provided support when needed, an overall sense of safety, and facilitated greater movement during game play by reducing the fear of falling.

Overall, the use of an assistive device during the session (ie, manual wheelchair or prosthetic limb) did not inhibit gaming activities. For participants who played seated in their manual wheelchair, blocks were placed behind their back wheels to keep them stationary for a better response to their movements. However, both participants who wore a prosthetic leg (one above the knee and one below the knee amputee) reported that they had to adjust their stance on the GAIMplank to obtain a better response.

The positioning of the external buttons and variable trigger used for game play actions such as jumping, shooting, and car acceleration was acceptable for all participants. Participants reported that the ability to adjust the height of the buttons and position them relative to their arm reach and preferred side (right, left) facilitated game play. The participants felt that the size of the buttons was suitable and that they were easy to access. One participant, who had difficulty keeping his hand on the buttons because of partial paralysis, reported that the surface friction of the buttons could be improved.

#### Usability of the GAIMplank

This category describes the participants’ perceptions regarding the use of the GAIMplank controller for playing video games. Specific aspects that were discussed included learning how to move for game control and responsiveness of the GAIMplank. Participants across the 3 groups acknowledged that it took some time to learn how to control and shift their weight to maneuver their character in the game. Sometimes it took time for them to feel secure to move.

Some participants reported inconsistency issues with the GAIMplank not calibrating correctly and not being responsive to their weight shifts. In some instances, recalibrating the system helped. Despite technical issues with the board during some sessions, the participants felt positive and eager to continue playing. Regardless of responsiveness issues with the GAIMplank, several participants indicated that this was the first time they were able to play such games because of accessibility issues with other AVG systems.

#### Overall Experience Using the GAIMplank for Video Game Play

Participants described their overall experience using the GAIMplank for video game play. Participants mainly shared positive feedback regarding their experiences with the device. As noted above, the most frequently reported barrier affecting the overall experience was the inconsistency in responsiveness of the GAIMplank system. Specific aspects highlighted by the participants regarding the GAIMplank were that the device is innovative, playing video games using the GAIMplank increases the physical activity demands of videogaming, and using the device is enjoyable. The participants expressed an interest in participating in future AVG research projects and having a device like the GAIMplank in their home setting.

#### “Active” Video Gaming

Several participants noted that playing video games using the GAIMplank system provided a level of physical activity. Participants described how they enjoyed the experience of moving their body to play the games instead of sitting on the couch and simply using a handheld controller or their phone. Although some frustration was experienced when the controller did not respond as expected to their movements, the participants acknowledged the value of this accessible controller in allowing them to engage in AVG play, which most of the participants were unable to do with current OTS products. Description of their game play experience included statements such as the following:

I like the board because I get to move with my body versus just sitting down. Gets me up instead of just using phone. This gaming board encourages body movement.

AVGu011, walks without an assistive device, played standing

It was fun. It was different. With me I’m reserved to buy some of the more active games, not knowing if I can do them. To come to a controlled environment and be able to do the movements, able to sense the movements with a prosthetic.

AVGu017, walks with a prosthetic leg, played standing

Use the whole body. It would be great to know how many calories burned.

AVGu013, uses a manual wheelchair for mobility, played in a wheelchair

But at the same time its accomplishing different goals, its being more core strength and stuff than with a (hand) controller and so...(shoulder shrug).

AVGu014, uses a manual wheelchair for mobility, played in a wheelchair

#### Future Iterations

The participants were asked to provide suggestions for improving the GAIMplank and any additional features they would like to see incorporated. The input from standing players included visual markers for foot placement, inward adjustment of handrails, handrail hook–like grip to accommodate persons without dexterity, and elevated placement of the LED matrix for better visibility. For individuals who were seated in their wheelchair, a suggestion was made to eliminate the use of the blocks behind the back wheels by adding some sort of ridge or divot to keep them from rolling.

### Researcher Observations

During data collection for several participants (approximately 50%), technical difficulties with the GAIMplank controller occurred. The most frequent were calibration issues. During these sessions, the calibration procedure did not work as intended resulting in an inability of the device to find center, and causing difficulty for players to produce the necessary actions for game play. Recalibration and adjustments to the sensitivity and bias were attempted, but the problem was not corrected. Adjustments to the participant’s positioning on the board were also attempted, but these were not successful. Sometimes it was just one particular game during a session that would not respond to the participant’s movement. A pattern to these difficulties was not detected, and usually during the next testing session the controller worked appropriately. Specific issues based on play style (wheelchair, seated in a 4-legged chair, or standing) were not evident, except for participants who played standing and were not able to distribute their weight evenly. It appeared that the calibration procedure may not have accounted for this shift in body weight. Owing to time constraints, sufficient time was not always available to spend on troubleshooting during participant visits. Although enjoyment and RPE scores were likely affected by technical issues, participants still reported having fun and felt confident in their ability to use the system. Participants also recognized the system as providing an opportunity, typically not available to them, to engage in AVG.

## Discussion

### Principal Findings

Physical activity options are limited for people with mobility impairments. AVG, also known as exergaming, has the potential to provide a fun and engaging way to increase daily physical activity or reduce sedentary time. This project aimed to develop an AVG controller that was accessible and usable by individuals with mobility impairments. A sample of 21 adults with various mobility impairments was able to successfully access and play a series of standard PC video games using trunk and body movements to produce game play action. Participant usability scores (SUS and HITUES) and qualitative feedback indicated above average usability for the GAIMplank system. A previous study adapted the Wii Fit balance board controller and demonstrated successful use among people with mobility impairments with an average SUS score of 71.7 (SD 18.03) [17]. Physiological testing during game play on the adapted board indicated light-to-moderate intensity exercise for some participants [18]. This previously adapted gaming board controller was limited to use with only Wii Fit games, whereas the new GAIMplank system includes several new options for playing AVGs among people with mobility impairments and allows use with all PC games. The GAIMplank system provides a means for making games that are typically sedentary, using only the hands and fingers, more active by incorporating larger body movements. Furthermore, participants reported game play enjoyment and for some their first opportunity to engage in AVG owing to accessibility issues with OTS systems.

Looking at the average SUS scores for each participant, the lowest score was 40, well below what is considered acceptable usability. Similarly, the participant’s HITUES score (4.18) suggested below acceptable usability. The participant was a regular video game player (120 minutes per week), played seated in his wheelchair, reported moderate level enjoyment across games, game play was rated as a light-intensity exercise, and a large drop in self-efficacy was reported at the end of the session for the questions that pertained to reacting fast enough to choose the next action and moving well enough to maintain successful play. His postplay feedback highlighted issues with responsiveness, which he found frustrating. The next lowest SUS score (52.5) was also reported by a wheelchair user, which can be attributed to the GAIMplank malfunctioning, making the calibration off and the games moving slow. Regardless, this participant who was physically active (960 minutes per week) and a frequent video game player (320 minutes per week) reported above average enjoyment scores, game play as light-intensity exercise, with an improvement in overall self-efficacy after using the GAIMplank system. Of the 4 other participants who reported less than optimal usability (62.5-65), 3 (75%) were standing players who were all physically active, with varying levels of weekly video game play. Two players also had low HITUES scores (4.15) and reported moderate enjoyment, light-intensity exercise, and above average self-efficacy. In both cases, technical issues were encountered with the GAIMplank system during the testing session, resulting in poor responsiveness and making the games very difficult to play. The other standing player typically used a rollator to assist with mobility but was able to play without it. His average enjoyment score was high (86) with light-intensity RPE ratings and maximum self-efficacy scores. Although his SUS score was low (62.5), his HITUES score (4.8) was the highest among all participants. These scores may suggest that although the participant perceived general usability (SUS) issues with the system, he recognized the usability of the GAIMplank as an adapted controller to provide an option for active video game play for individuals with mobility impairments.

At the other end of the spectrum, 7 players reported “excellent” usability (SUS ≥85) for the GAIMplank system. Of those, 2 played in their wheelchair, 2 played standing, and 3 played while seated in the chair. For the standing and seated players, the HITUES scores reflected acceptable usability. All 3 seated players (poststroke) spent little to no time playing video games each week but were physically active, with 2 reporting >400 minutes per week of physical activity. The average enjoyment scores were high, the RPE scores varied, and all were reported to have certainty in their ability to conduct various video game play tasks using the GAIMplank system. Both standing participants were physically active (120 minutes per week); one spent a lot of time playing video games each week (630 minutes), whereas the other played very little, if any. Average enjoyment scores were higher for the person who typically spent a lot of time playing video games each week, whereas RPE was higher for the other participant. Both participants reported high self-efficacy in using the GAIMplank system for video game play. Both participants who played seated in their wheelchairs with high SUS scores were physically active (120 minutes per week); one spent a lot of time playing video games each week (210 minutes), whereas the other one played none. The HITUES scores were lower than those of other participants who reported high SUS scores. Both players enjoyed the games, with RPE scores indicating light-to-moderate intensity exercise. The self-efficacy scores were high for both players at the end of the session.

The results demonstrate the usability of a newly developed adapted gaming board for AVG play, but attention is needed to improve the stability of responsiveness and to address issues experienced by different subgroups of individuals with mobility impairments. Considerations for the engineering team during the next phase of development include different weight distribution patterns during game play by individuals wearing a prosthetic leg, varying movements associated with spasticity among certain players, foot placement outside the board (ie, on stool in front) for some seated players, and overall improvement to better accommodate wheelchair play. Overall, the ability of the adapted controller to provide a fun and accessible option for AVG play was clearly recognized by the players, suggesting the need for additional development and research in this area.

### Study Limitations

The heterogeneous nature of the sample makes it difficult to determine whether different aspects of the GAIMplank better suited one group over another. The number of participants within each play style group was small and consisted of individuals with various mobility impairments. In addition, participants were recruited from a community-based health and fitness center, so all had experience with physical activity and were currently active with reported weekly physical activity minutes ranging from 60 to 960 (mean 375, SD 257) minutes. Therefore, the results cannot be generalized to more sedentary populations. Previous gaming experience was captured, but its influence on system usability or their game play experience could not be teased out. With regard to the specific games used for the sessions, game selection was limited and may not have appealed to some participants. In addition, sufficient time to learn how best to move for each game was not provided. Initially, qualitative feedback data were not collected from participants. As the need for more in-depth user input was recognized, semistructured interviews were conducted to collect richer feedback data. This change occurred after most of the seated players had completed testing; therefore, limited qualitative data were available from this group.

### Future Considerations

Continued testing and refinement of the GAIMplank system are underway. The usability testing results were discussed with the engineering and design teams, and a new wave of refinements is planned with a focus on improving the stability of the platform. Specifically, elimination of the Raspberry Pi and addition of a custom-fabricated PCB board are being considered. Following further refinement of the system, the plan is to conduct a feasibility study to examine the use of the GAIMplank for an AVG exercise intervention. The outcomes of interest will include various physiological and psychosocial measures. Other factors to be considered include game play selection and preferences, previous gaming experience, participants’ physical activity level, and single versus multiplayer options. In addition, consideration of factors that affect game play-by-play style (eg, seated and standing) needs to be examined. The potential of integrating other gaming controllers is also of interest.

### Conclusions

The GAIMplank, an adapted gaming controller, allowed people with various mobility impairments to engage in AVG. Using the GAIMplank system, standard PC video games can be played while sitting, standing, and in a manual wheelchair. Participants reported above average usability for the GAIMplank and found game play to be enjoyable and provide light-to-moderate intensity exercise. Further design and development work to refine the device is recommended.

## Appendix

Multimedia Appendix 1 Survey to assess participant pregame and postgame play self-efficacy.

Multimedia Appendix 2 Health-IT Usability Evaluation Scale used to collect usability data specific to active video gaming using the GAIMplank.

Multimedia Appendix 3 Survey questions used to gather additional feedback from participants regarding the GAIMplank.

Multimedia Appendix 4 Questions used to guide semistructured interview with participants to collect feedback data at the end of game play using the GAIMplank system.

## Abbreviations

AVG: active video gaming
HITUES: Health-IT Usability Evaluation Scale
OTS: off-the-shelf
PCB: printed circuit board
PoC: proof-of-concept
PoP: proof-of-product
RPE: rating of perceived exertion
SUS: System Usability Scale
