# Supplementary material for: Usability of the GAIMplank Video Game Controller for People With Mobility Impairments: Observational Study
Source: JMIR Serious Games. 2023 Jan 10;11:e38484. doi: 10.2196/38484 (PMC9947916; doi:10.2196/38484)
Supplement: Multimedia Appendix 1 [file games_v11i1e38484_app1.docx]

**PRE-Play Video Game Play Task Self-Efficacy**

Now that we have explained how you will play video games using the **Adapted Board**, please indicate how certain or uncertain you are regarding each of the video game play aspects listed below.

*Rate your degree of certainty by recording a number from 0 to 10 using the scale provided.*

0 1 2 3 4 5 6 7 8 9 10
No Moderate Absolute
certainty certainty certainty

**Certainty (0 to 10)**

Maintaining focus for at least 5-minutes ________________

Seeing and hearing all of the game information ________________

Reacting fast enough to choose a next action ________________

Determining strategies to move during play ________________

Coordinating body movements to carry out a strategy ________________

Moving well enough to maintain successful play ________________

**POST-Play Video Game Play Task Self-Efficacy**

Now that we have explained how you will play video games using the **Adapted Board**, please indicate how certain or uncertain you are regarding each of the video game play aspects listed below.

*Rate your degree of certainty by recording a number from 0 to 10 using the scale provided.*

0 1 2 3 4 5 6 7 8 9 10
No Moderate Absolute
certainty certainty certainty

**Certainty (0 to 10)**

Maintaining focus for at least 5-minutes ________________

Seeing and hearing all of the game information ________________

Reacting fast enough to choose a next action ________________

Determining strategies to move during play ________________

Coordinating body movements to carry out a strategy ________________

Moving well enough to maintain successful play ________________
