# Supplementary material for: Usability of the GAIMplank Video Game Controller for People With Mobility Impairments: Observational Study
Source: JMIR Serious Games. 2023 Jan 10;11:e38484. doi: 10.2196/38484 (PMC9947916; doi:10.2196/38484)
Supplement: Multimedia Appendix 2 [file games_v11i1e38484_app2.docx]

**Adapted Gaming Board**

**Health-ITUES**

|  | **Strongly Agree** |  |  |  | **Strongly Disagree** |
| --- | --- | --- | --- | --- | --- |
| **IMPACT** |  |  |  |  |  |
| 1. I think the *Adapted Gaming Board* would be a positive addition for persons with mobility impairments. | **5** | **4** | **3** | **2** | **1** |
|  |  |  |  |  |  |
| 2. I think the *Adapted Gaming Board* would provide a physical activity option for persons with mobility impairments. | **5** | **4** | **3** | **2** | **1** |
|  |  |  |  |  |  |
| 3. The *Adapted Gaming Board* would be an important part of meeting my physical activity needs. | **5** | **4** | **3** | **2** | **1** |
| **PERCEIVED USEFULNESS** |  |  |  |  |  |
| 4. Using the *Adapted Gaming Board* makes it easier to play (active) video games. | **5** | **4** | **3** | **2** | **1** |
|  |  |  |  |  |  |
| 5. Using the *Adapted Gaming Board* enables me to be more active when playing video games. | **5** | **4** | **3** | **2** | **1** |
|  |  |  |  |  |  |
| 6. Using the *Adapted Gaming Board* makes it more likely that I will engage in active video gaming. | **5** | **4** | **3** | **2** | **1** |
|  |  |  |  |  |  |
| 7. Using the *Adapted Gaming Board* is useful for being more active during video game play. | **5** | **4** | **3** | **2** | **1** |
|  |  |  |  |  |  |
| 8. I think the *Adapted Gaming Board* presents a more equitable process for engaging in active video gaming. | **5** | **4** | **3** | **2** | **1** |
|  |  |  |  |  |  |
| 9. I am satisfied with the *Adapted Gaming Board* for engaging in active video gaming. | **5** | **4** | **3** | **2** | **1** |
|  |  |  |  |  |  |
| 10. I engage in active video gaming in a timely manner because of the *Adapted Gaming Board*. | **5** | **4** | **3** | **2** | **1** |
|  |  | | | | |
| 11. Using the *Adapted Gaming Board* increases my ability to engage in active video gaming. | **5** | **4** | **3** | **2** | **1** |
|  |  | | | | |

**Adapted Gaming Board**

**Health-ITUES**

|  | **Strongly Agree** |  |  |  | **Strongly Disagree** |
| --- | --- | --- | --- | --- | --- |
|  |  | | | | |
| 12. I am able to engage in active video gaming whenever I use the *Adapted Gaming Board*. | **5** | **4** | **3** | **2** | **1** |
| **PERCEIVED EASE OF USE** |  | | | | |
| 13. I am comfortable with my ability to use the *Adapted Gaming Board*. | **5** | **4** | **3** | **2** | **1** |
|  |  | | | | |
| 14. Learning to operate the *Adapted Gaming Board* is easy for me. | **5** | **4** | **3** | **2** | **1** |
|  |  | | | | |
| 15. It is easy for me to become skillful at using the *Adapted Gaming Board*. | **5** | **4** | **3** | **2** | **1** |
|  |  | | | | |
| 16. I find the *Adapted Gaming Board* easy to use. | **5** | **4** | **3** | **2** | **1** |
|  |  | | | | |
| 17. I can always remember how to log on to and use the *Adapted Gaming Board*. | **5** | **4** | **3** | **2** | **1** |
| **USER CONTROL** |  | | | | |
| 18. The *Adapted Gaming Board* gives error messages that clearly tell me how to fix problems. | **5** | **4** | **3** | **2** | **1** |
|  |  | | | | |
| 19. Whenever I make a mistake using the *Adapted Gaming Board*, I recover easily and quickly. | **5** | **4** | **3** | **2** | **1** |
|  |  | | | | |
| 20. The information (e.g., on-screen messages, other documentation) provided with the *Adapted Gaming Board* is clear. | **5** | **4** | **3** | **2** | **1** |
|  |  | | | | |
