# Supplementary material for: Usability of the GAIMplank Video Game Controller for People With Mobility Impairments: Observational Study
Source: JMIR Serious Games. 2023 Jan 10;11:e38484. doi: 10.2196/38484 (PMC9947916; doi:10.2196/38484)
Supplement: Multimedia Appendix 3 [file games_v11i1e38484_app3.docx]

**Usability Feedback Survey Questions – Adapted Gaming Board**

1. Rate the ease of mounting the **Gaming Board**?
   1. Relatively Simple
   2. Needs improvement
   3. Difficult
2. Rate the ease of dis-mounting the **Gaming Board**?
   1. Relatively Simple
   2. Needs improvement
   3. Difficult
3. Did you feel the **Gaming Board** was sturdy? YES / NO
4. Were you able to determine where best to position yourself on the **Gaming Board**? YES / NO
   1. Should we include visual cues to locate the central position? YES / NO
5. Did moving your trunk (leaning) provide a responsive input for game control? YES / NO
6. What if any additional input functions would you like to see incorporated into the **Gaming Board**?
7. How was your overall experience on the **Gaming Board**?
