# Supplementary material for: Usability of the GAIMplank Video Game Controller for People With Mobility Impairments: Observational Study
Source: JMIR Serious Games. 2023 Jan 10;11:e38484. doi: 10.2196/38484 (PMC9947916; doi:10.2196/38484)
Supplement: Multimedia Appendix 4 [file games_v11i1e38484_app4.docx]

**Usability Semi-Structured Interview – Adapted Gaming Board**

*Thanks for participating today. We’d like to discuss certain features of each system with you. Please describe your experience in as much detail as you can.*

**Adapted Board**

1. Please describe your ease and/or difficulty of mounting/dismounting the gaming board?
2. Did your assistive device inhibit gaming activities? What if anything did you do to compensate?
3. Please describe your ease and/or difficulty of playing games using the gaming board?
4. Please describe your ease and/or difficulty of using accessory buttons for game play (e.g., jump, shoot, accelerate)
5. Did you use the handrails during gameplay? How did they contribute to your game play?
6. Describe whether or not moving your trunk (leaning) provided a responsive input for game control?
7. What if any additional features or functions would you like to see incorporated into the gaming board?
8. How was your overall experience using the gaming board?
9. Any other comments or feedback?
